# Hybrid Sequential Meniscal Suture Technique

**DOI:** 10.1002/atn2.70265

**Published:** 2026-10-04

**Authors:** Jose Leonardo Rocha de Faria, Igor Araujo Farias, Rafael Erthal de Paula, Sandra Tie Nishibe Minamoto, Diego Ariel, Yuri Di Cavalcanti Sampaio, Naasson Trindade Cavanellas, João Antonio Matheus Guimarães, Geraldo da Rocha Motta, José Paulo Gabbi Aramburú Filho, Camilo Partezani Helito, Phelippe Valente Maia

**Affiliations:** ^1^ National Institute of Traumatology and Orthopedics of Brazil Rio de Janeiro Rio de Janeiro Brazil; ^2^ School of Medicine from University of São Paulo São Paulo Brazil; ^3^ Universidade Federal Rural do Semi‐Árido ‐ UFERSA Mossoró Rio Grande do Norte Brazil

## Abstract

Meniscal preservation is a priority in the treatment of knee injuries because of the fundamental role of the meniscus in load absorption, joint stability, and chondral protection. Several repair techniques have been described, including inside‐out, outside‐in, and all‐inside approaches. More recently, continuous suturing techniques have emerged as a promising alternative, allowing multiple fixation points with shorter operative time and reduced costs. We describe a hybrid continuous meniscal repair technique that combines initial all‐inside fixation with additional outside‐in sutures using the excess suture limb from the implant. The repair begins with meniscal fixation using an all‐inside device, after which the free suture end is redirected percutaneously through successive outside‐in passages, creating a continuous vertical suture construct. This strategy enables multiple fixation points from a single device, potentially enhancing biomechanical stability and reducing the need for additional implants. The integration of all‐inside and outside‐in fixation allows the entire extent of the tear to be addressed in a safe, reproducible, and cost‐effective manner. The described hybrid technique expands the applicability of continuous meniscal suturing and may contribute to improved clinical outcomes in meniscal preservation.

## Graphical abstract

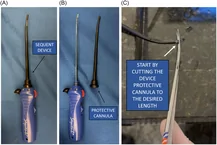

**VIDEO 1:** The technique is performed using the Sequent meniscal repair device for sequential all‐inside suturing. The protective cannula is trimmed to the desired length. Arthroscopy reveals a longitudinal medial meniscal tear at the posterior horn‐body junction. In the first step, we pierce the meniscus and deploy the initial fixation implant. From the second step onward, we follow the PRDT sequence, which includes 4 essential maneuvers:•P—Pierce: The initial meniscal penetration.•R—Rotate: The device is rotated twice, with two 180° turns.•D—Deploy: The implant is then deployed into the meniscal tissue.•T—Tension: Finally, the suture is tensioned to secure the repair.It is important to maintain a small residual loop, avoiding excessive tightening within the joint. These steps can be repeated as needed, depending on the number of available implants and the extent of the tear, always following the PRDT sequence. At this stage, the suture is typically cut. However, in this hybrid technique, the same suture is used to continue the repair with an outside‐in approach. A transport needle, such as a spinal or skin needle, is introduced, and the Sequent suture is passed through the created loop. The loop is then retrieved outside the joint, as shown in this anatomical model. Next, the needle is introduced through a second entry point, always crossing the meniscal tear. The loop is tensioned, and the suture is passed through it, creating a second loop outside the joint. These loops should be consistently maintained and organized outside the joint. In the third step, the same process is repeated, and the free suture end is fully retrieved outside the joint. In this manner, a sequential meniscal repair is achieved using a single continuous suture construct. In this case, a total of 6 fixation points were placed to close an extensive longitudinal tear. To complete the repair, the free suture end is passed through the intermediate loop, corresponding to the second loop, and tension is applied to reduce the construct. This step must always be performed under direct arthroscopic visualization, ensuring proper tension and alignment. Finally, multiple knots are tied between the free suture end and the first loop, completing the fixation. The final arthroscopic view shows excellent tear reduction and stable meniscal repair. The procedure is now complete. Video content can be viewed at https://doi.org/10.1002/atn2.70265.

Meniscal tears are among the most common knee injuries and represent a frequent cause of functional disability.^1^, ^2^ The meniscus plays crucial roles in shock absorption, load transmission, and secondary stabilization of the joint.^2^, ^3^ Although meniscectomy may relieve early symptoms, it is associated with increased tibiofemoral contact pressures and the premature development of osteoarthritis.^2^, ^4^ Consequently, meniscal preservation has become a treatment priority, reflected in the contemporary movement toward meniscal repair.^1^, ^5^, ^6^, ^7^

Meniscal repair may be performed using inside‐out, outside‐in, or all‐inside techniques.^3^, ^8^, ^9^, ^10^ The choice depends on tear pattern, location, and surgeon experience.^2^ Comparative studies show that repair preserves long‐term joint function more effectively than meniscectomy, including in high‐level athletes.^2^, ^4^, ^11^ In this context, the outside‐in technique is particularly useful for tears in the meniscal body and anterior horn, whereas all‐inside repair offers reduced surgical morbidity and shorter operative time, but at a higher cost.^8^, ^12^

The inside‐out technique remains the gold standard because of its versatility and ability to place multiple sutures in different meniscal regions.^8^, ^13^, ^14^ However, it has relevant limitations, such as additional incisions and risk of neurovascular complications.^8^, ^13^, ^14^ Modern all‐inside devices have expanded access to complex tears, but high cost and implant‐related failures remain critical concerns.^2^, ^9^, ^12^

Recently, continuous suturing techniques have been introduced to optimize meniscal repair. Experimental studies have shown that vertical continuous suturing provides greater operative efficiency and a shorter learning curve while maintaining adequate biomechanical stability.^13^, ^14^, ^15^ The ability to place multiple fixation points using a single device reduces costs and expands clinical applicability, especially for extensive longitudinal tears in the peripheral meniscus.^4^, ^13^, ^15^, ^16^

In this scenario, combining all‐inside implants with sequential outside‐in suturing using the excess suture limb represents a promising alternative. This hybrid approach may increase the number of fixation points, enhance repair stability, and reduce costs. The purpose of this technical note is to describe a sequential meniscal suturing technique integrating an all‐inside device with the outside‐in approach, providing a reproducible and economically viable solution for meniscal preservation.

## SURGICAL TECHNIQUE

Readers are invited to watch the accompanying video for a detailed step‐by‐step illustration of the surgical technique. The patient is positioned supine under general or spinal anesthesia, according to anesthetic evaluation. After antiseptic preparation and placement of a pneumatic tourniquet at the proximal thigh, the procedure is performed with the limb hanging over the edge of the table, allowing full joint mobility. Two anterior arthroscopic portals are used: an anterolateral and an anteromedial portal.

After diagnostic arthroscopy and confirmation of a longitudinal meniscal tear, the Sequent device (CONMED, Utica, NY), developed for sequential meniscal repair, is introduced through the working portal (Figure 1A‐C). In this technique, 4 sequential all‐inside fixation points are placed to approximate the torn meniscal edges, completing 3 meniscal sutures. After the fourth fixation point, the excess suture limb remains free. This suture is then used to continue the repair with an outside‐in technique. For this purpose, a 16‐gauge epidural needle is preloaded with a shuttle loop. The needle is introduced percutaneously through the medial aspect of the knee along the tear trajectory, crossing the capsule and meniscus (Tables 1 and 2).

**FIGURE 1:**
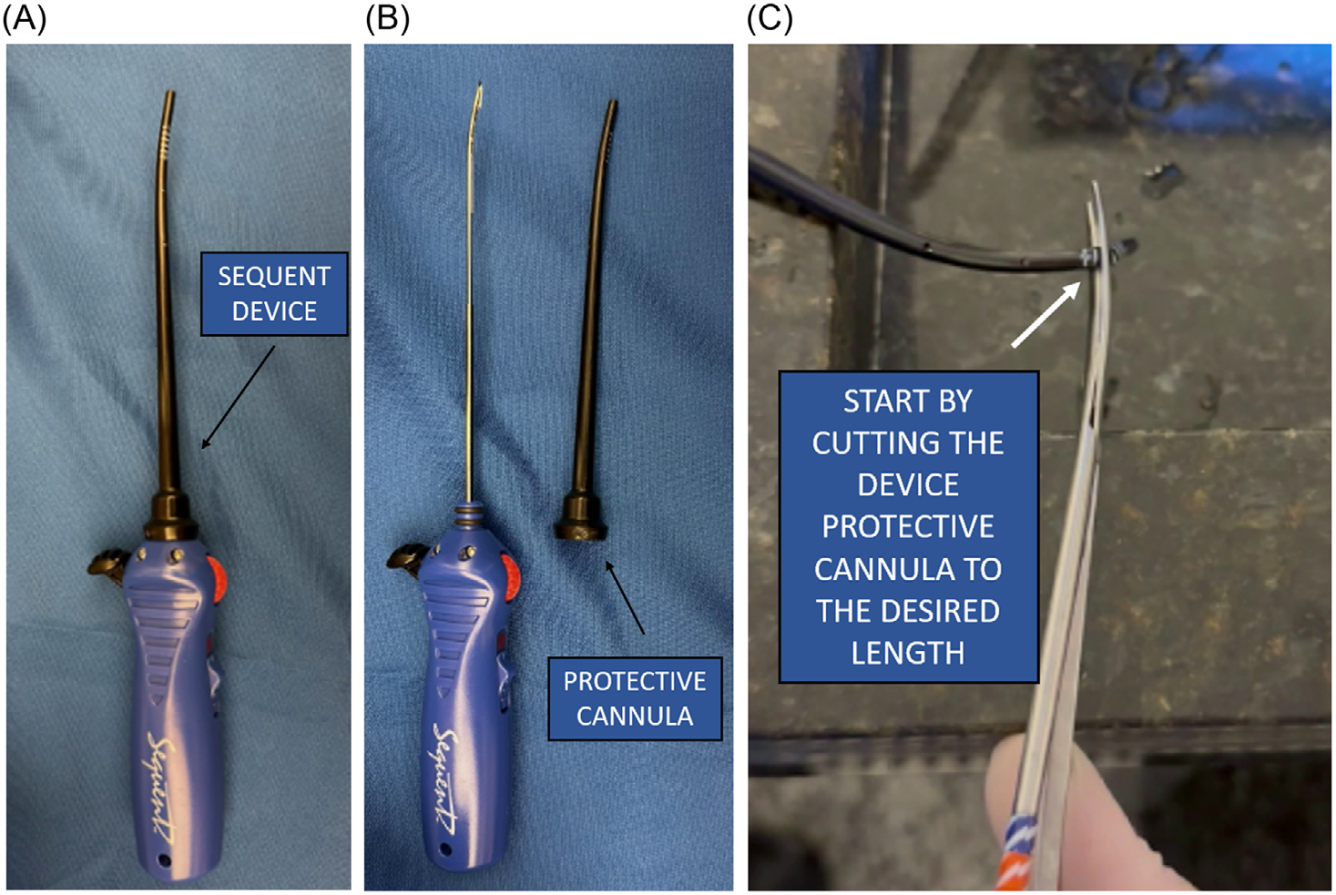
(A,B) All‐inside meniscal repair device with 4 rectangular implants, allowing 3 sequential fixation points. (C) The protective cannula is trimmed at the 16 mm mark, allowing the device tip to penetrate the meniscus to a depth of 16 mm.

**TABLE 1:** Advantages and Disadvantages

Advantages	Disadvantages
Enables sequential repair using a single continuous suture, reducing the need for multiple independent sutures	Requires a learning curve for correct execution of PRDT steps and outside‐in suture handling
Combines all‐inside and outside‐in approaches, expanding reach in difficult tear locations	May increase operative time in early cases because of loop organization and traction
Uses a single device (Sequent), reducing instrument exchange and simplifying intraoperative workflow	Risk of loop confusion if extracapsular loops are not consistently organized
The PRDT sequence standardizes steps, improving reproducibility	Requires precise arthroscopic visualization to tension loops adequately, especially in extensive tears
Allows closure of extensive longitudinal tears with multiple sequential fixation points	Requires careful tension management to avoid excessive meniscal compression

PRDT, Pierce, Rotate, Deploy, Tension.

**TABLE 2:** Pearls and Pitfalls

Pearls	Pitfalls
Always maintain a small residual loop after each tensioning step	Poor extracapsular loop organization may lead to entanglement and loss of suture control
Use the PRDT acronym to maintain a consistent sequence	Over‐ or under‐rotation may compromise implant fixation
Retrieve each loop extracapsularly while maintaining a clear arthroscopic field	Pulling without arthroscopic visualization may result in inadequate tension or suture failure
Confirm implant and loop positioning before definitive tightening	Excessive tension may cause posterior implant loosening and migration into the joint
Monitor loop tension under direct visualization to ensure proper tear reduction	Ignoring meniscal biomechanics may result in overtightening or malpositioned sutures

PRDT, Pierce, Rotate, Deploy, Tension.

In the first step of the technique, the device pierces the meniscus and deploys the initial fixation implant (Figure 2A,B). From the second step onward, the PRDT sequence is performed (Figure 3A‐D):

• P—Pierce: Initial meniscal penetration.
• R—Rotate: The device is rotated twice, with two 180° turns.
• D—Deploy: The implant is deployed into the meniscal tissue.
• T—Tension: The suture is tensioned to secure fixation.

**FIGURE 2:**
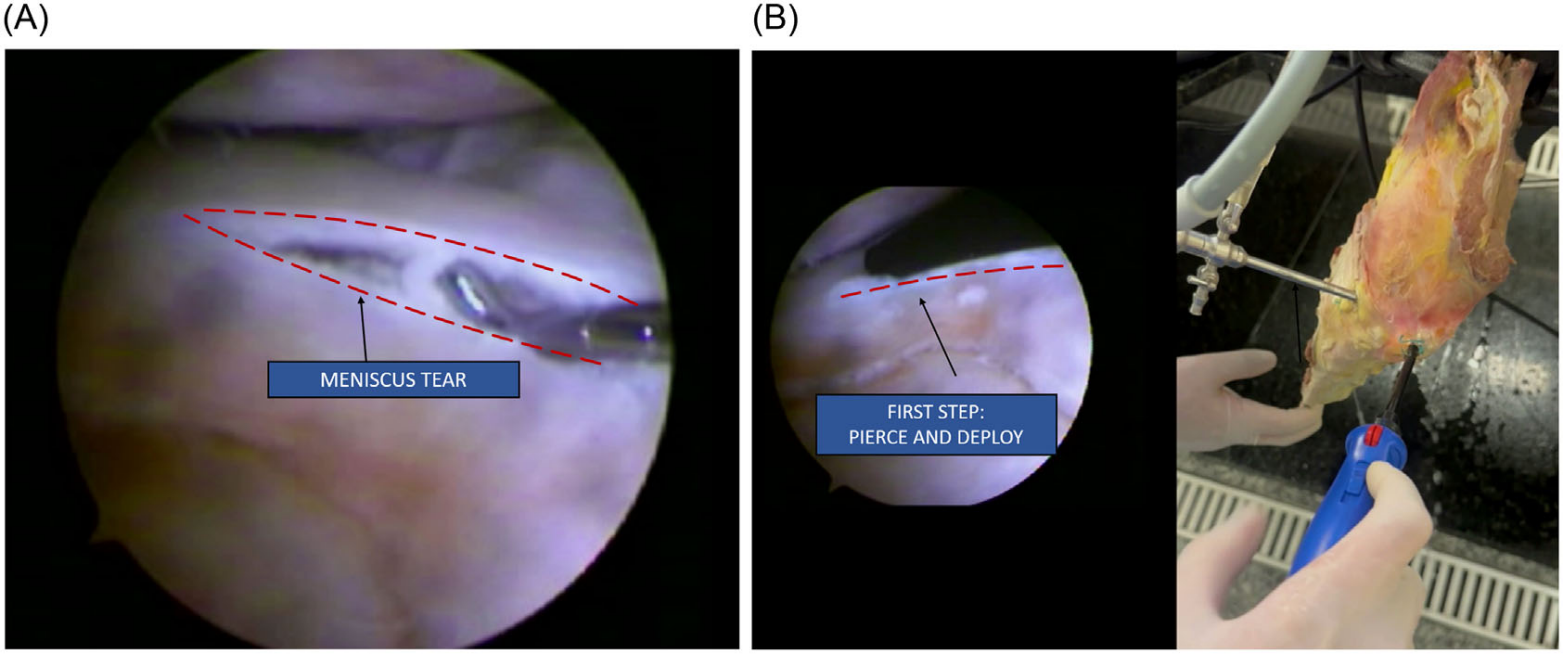
(A) Arthroscopic view showing a longitudinal tear at the posterior horn‐body junction of the medial meniscus. (B) The meniscal repair device penetrates proximal to the tear, and after full advancement of the Sequent tip, the first implant is deployed by pressing the release button on the device.

**FIGURE 3:**
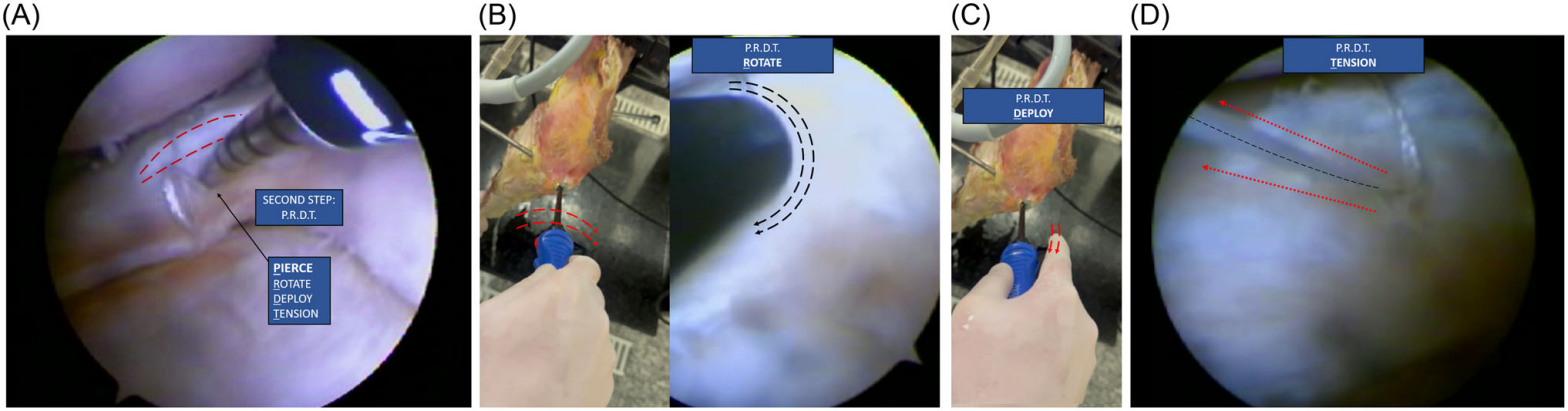
(A) From the second step onward, the tear is crossed obliquely and the PRDT sequence is followed. P—Pierce: Initial meniscal penetration. (B) R—Rotate: The device is rotated twice, with two 180° turns. (C) D—Deploy: The implant is deployed into the meniscal tissue. (D) T—Tension: The suture is tensioned to secure the repair. (PRDT, Pierce, Rotate, Deploy, Tension.)

It is important to maintain a small residual loop, avoiding excessive tightening within the joint (Figure 4A‐C).

**FIGURE 4:**
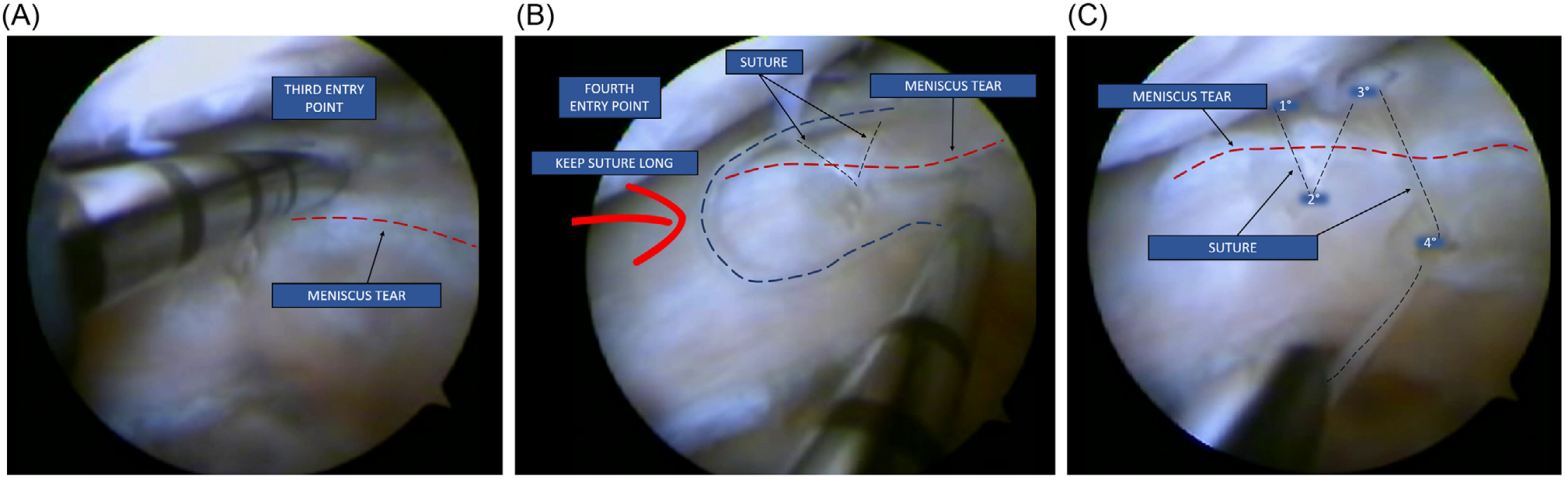
(A) For the third fixation point, the tear is again crossed obliquely and the PRDT steps are repeated. (B) On returning to the joint to initiate the next fixation, it is important to leave a small intra‐articular suture loop, avoiding premature tensioning of the next implant during meniscal penetration. (C) Four distinct meniscal penetration sites are observed, where rectangular implants were deployed, creating 4 sequential oblique fixation points. (PRDT, Pierce, Rotate, Deploy, Tension.)

These steps can be repeated as needed, depending on the number of available implants and the extent of the tear, always following PRDT (Figure 3A‐D). At this stage, the suture is typically cut. However, in this hybrid technique, the same suture is preserved and used to continue the repair through an outside‐in approach.

A transport needle, such as a 16‐gauge epidural needle preloaded with a shuttle loop, is introduced, and the Sequent suture is passed through the created loop. The loop is then retrieved outside the joint, as shown in the anatomical model (Figure 5A‐G).

**FIGURE 5:**
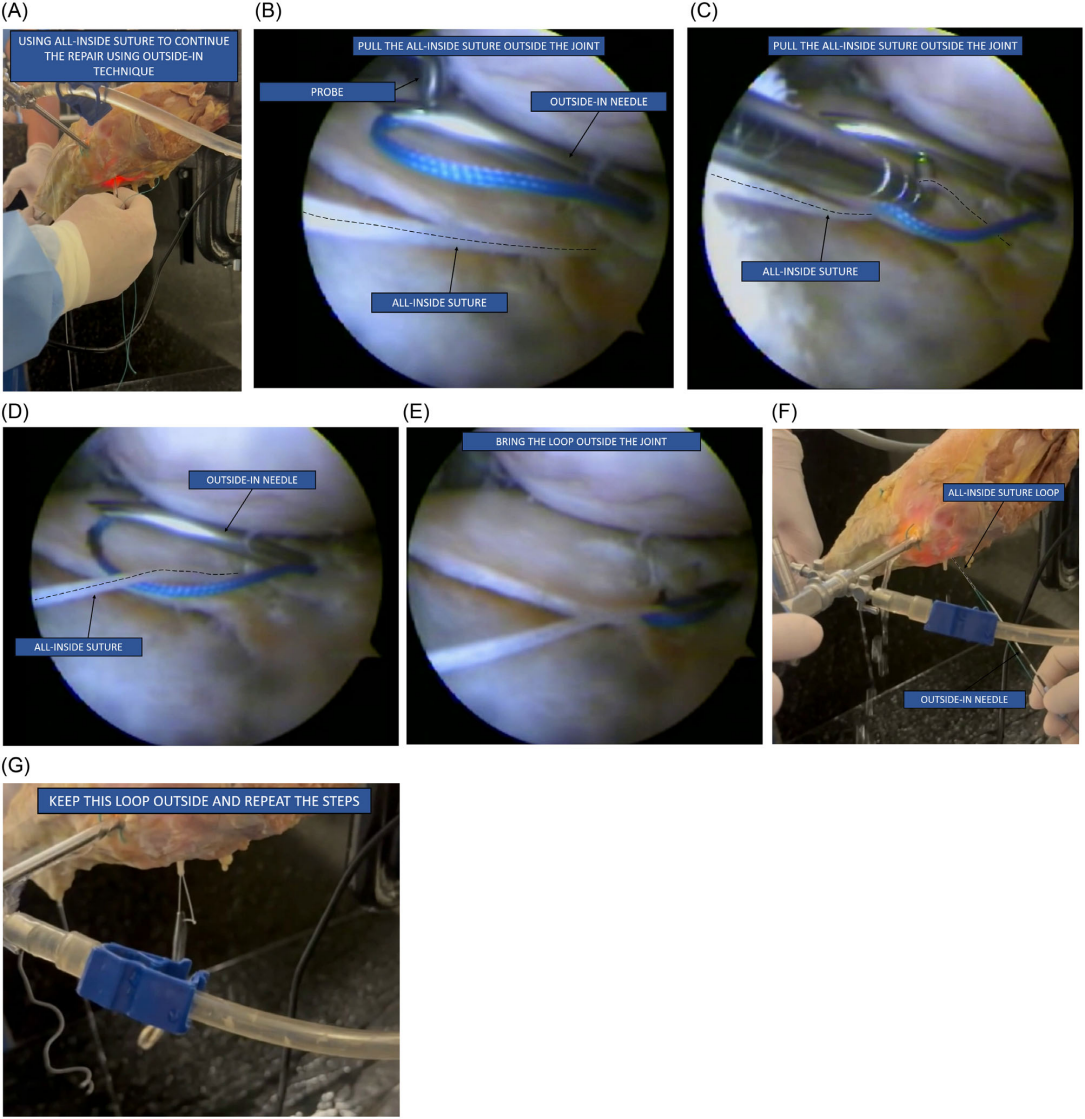
(A) The suture limb that is typically cut at this stage is instead preserved and used to continue the repair, being retrieved outside the joint using an outside‐in meniscal repair technique. (B) A 16‐gauge epidural needle prepared with an Ethibond suture loop is used to shuttle the Sequent suture limb outside the joint. The needle is introduced on the opposite side of the tear relative to the intra‐articular suture limb. (C,D) A probe is used to pass the Sequent suture limb through the Ethibond loop. (E‐G) The suture is pulled, forming an extracapsular loop outside the joint.

Next, the needle is introduced through a second entry point, always crossing the meniscal tear. The loop is tensioned, and the suture is passed through it, creating a second loop outside the joint (Figure 6A‐F). These loops should be consistently maintained and organized outside the joint.

**FIGURE 6:**
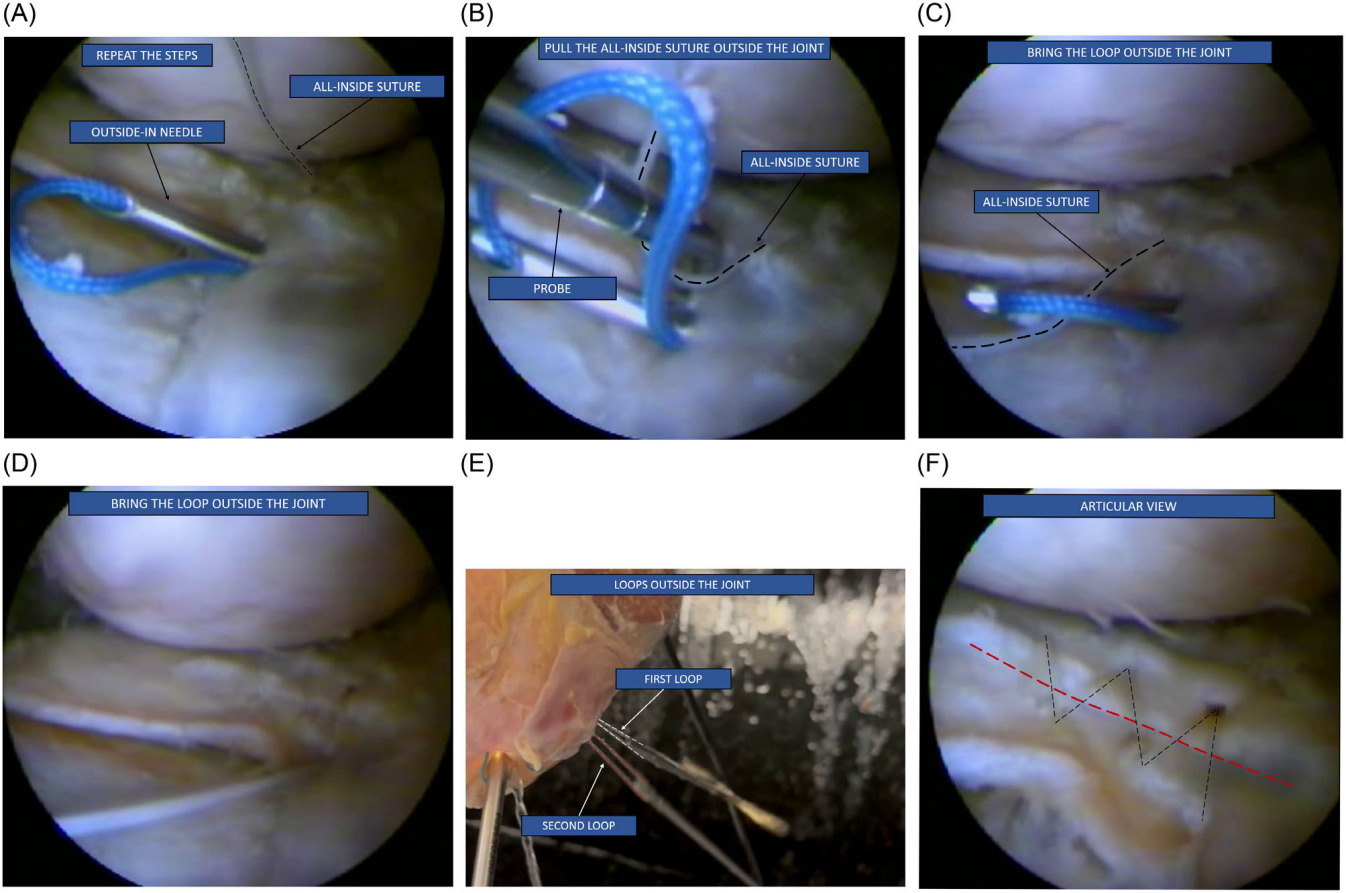
(A‐D) The steps described in Figure 5 are repeated, always introducing the epidural needle at a location opposite to the most recent Sequent suture penetration site. (E) The first extracapsular loop and the newly formed second loop are observed outside the joint. (F) At this stage, a total of 5 fixation points have been completed.

In the third step, the same process is repeated, and the free suture end is fully retrieved outside the joint. In this manner, a sequential meniscal repair is achieved using a single continuous suture construct. In this case, a total of 6 fixation points were placed to close an extensive longitudinal tear (Figure 7A,B).

**FIGURE 7:**
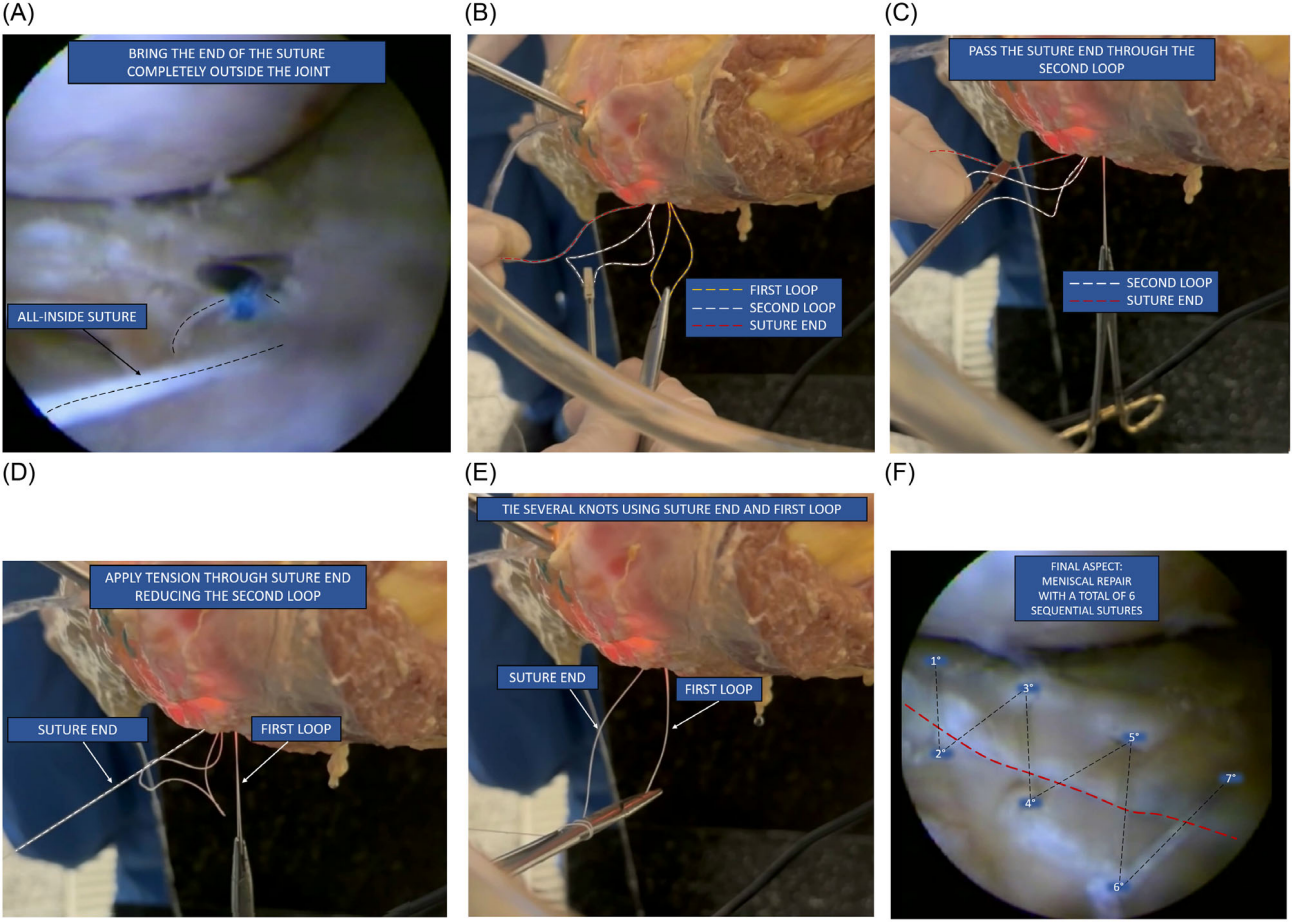
(A) The steps described in Figures 5 and 6 may be repeated as many times as necessary to repair the entire tear. To finalize the technique, the suture limb is fully retrieved outside the joint. (B) In this case, the first and second loops are formed, followed by complete retrieval of the free Sequent suture limb. (C,D) The free suture end is passed through the second (intermediate) loop, and traction is applied to reduce this loop. (E) Final fixation is achieved by tying 3–4 knots between the free suture end and the first loop, which is considered a single working strand. (F) Final arthroscopic view showing 6 fixation points securing the longitudinal meniscal tear.

To complete the repair, the free suture end is passed through the intermediate loop, corresponding to the second loop, and tension is applied to reduce the construct. This step must always be performed under direct arthroscopic visualization, ensuring proper tension and alignment (Figure 7C‐E). Finally, multiple knots are tied between the free suture end and the first loop, completing the fixation. The final arthroscopic view shows excellent tear reduction and stable meniscal repair (Figure 7F). The procedure is now complete.

A representative clinical case is shown to reveal how this approach can be applied in a patient. A small medial skin incision of only a few millimeters is made exclusively to externalize the loops (Video 1). At this point, the sutures are retrieved and knots are tied extracapsularly, thereby securing the meniscus.

This hybrid technique enables multiple continuous fixation points using a single all‐inside device, optimizing the use of the excess suture limb and reducing the need for additional implants. The supplementary outside‐in sutures allow more uniform load distribution along the entire tear, providing enhanced biomechanical stability. Final arthroscopic inspection confirms adequate stability and anatomic reduction of the meniscal tear (Figure 7F).

## DISCUSSION

The technique described in this study proposes a hybrid solution combining the initial security of an all‐inside device with the efficiency of continuous outside‐in suturing using the excess suture limb. This technical modification enhances biomechanical stability, increases the number of fixation points without additional implants, and maintains minimal morbidity because it requires only a small incision for final extracapsular knot tying. This approach aligns with current trends toward safer, cost‐effective repairs with simplified learning curves.^1^, ^15^

Meniscal preservation has become a paradigm in the treatment of knee injuries, supported by biomechanical, biological, and clinical evidence. Experimental studies show that meniscectomy, even when partial, increases tibiofemoral contact pressures, accelerates chondral degeneration, and promotes early osteoarthritis.^2^ From a biological standpoint, recent advances have identified resident progenitor cells in all meniscal zones, including regions traditionally considered avascular, along with more extensive vascularity than previously believed, indicating intrinsic healing potential.^4^ This body of evidence reinforces the philosophy of meniscal preservation guiding contemporary surgical practice.^1^

Long‐term reviews confirm that meniscal repair is associated with less progression of joint degeneration and better functional outcomes than meniscectomy.^2^, ^3^, ^17^ In professional athletes, meniscal preservation has shown higher return‐to‐sport rates and prolonged competitive longevity, whereas meniscectomy correlates with shortened career duration.^4^, ^18^ Nevertheless, meniscectomy continues to be performed disproportionately more often, reported as 5 to 25 times more frequent than repair, highlighting technical barriers and the need for broader dissemination of safe and reproducible repair techniques.^1^

Among available techniques, inside‐out repair remains the gold standard because it allows multiple sutures in different meniscal regions and provides reliable biomechanical strength.^13^, ^19^ However, it requires additional incisions and is associated with the risk of neurovascular injury.^8^, ^20^ The outside‐in technique is particularly useful for anterior tears, with reported failure rates below 6%.^8^ All‐inside devices have become popular because of ease of execution and lower morbidity, but limitations related to cost and implant‐related complications persist.^12^, ^21^

In this context, sequential or continuous techniques have emerged as a promising alternative. Continuous vertical inside‐out repair has shown adequate stability even for extensive longitudinal tears, with the advantage of multiple fixation points from a single construct.^14^ In experimental models, this technique significantly reduced operative time and showed a shorter learning curve than traditional vertical mattress suturing while maintaining biomechanical equivalence.^15^ In addition to technical efficiency, such methods contribute to cost rationalization, which is particularly relevant in resource‐limited health systems.^22^

In addition, our group has previously performed a controlled biomechanical study comparing continuous vertical inside‐out meniscal sutures with the traditional vertical mattress technique. In that investigation, no statistically significant differences were observed between the techniques regarding displacement after cyclic loading, construct stiffness, and ultimate load to failure, showing biomechanical equivalence between the continuous and conventional repair configurations. These findings support the mechanical reliability of continuous constructs and reinforce the rationale for extending this concept to hybrid techniques, such as the one described in the present study, in which multiple fixation points are achieved with a single suture construct while maintaining adequate biomechanical performance.^23^

Although grounded in solid biomechanical principles and prior experience with continuous and sequential suturing, this hybrid approach still lacks long‐term clinical studies and direct biomechanical comparisons with established techniques. Future investigations should evaluate failure rates, return‐to‐sport timelines, progression of chondral degeneration, and cost‐effectiveness across different populations. We believe that by optimizing available devices and integrating classic and modern concepts, this hybrid technique may represent a meaningful contribution to meniscal preservation and global advances in arthroscopic knee surgery.

## DISCLOSURES

The authors (J.L.R.F., J.P.G.A.F., C.P.H. P.V.M.) declare the following financial interests/personal relationships which may be considered as potential competing interests: J.L.R.F. reports personal fees from Síntegra Surgical Sciences, outside the submitted work, and personal fees from ConMed and has a patent Meniscal Suture Device N° US 11,589,861 B2 ‐ Meniscus 4ALL with royalties paid. J.P.G.A.F. reports personal fees from ConMed. C.P.H. reports personal fees from Smith & Nephew, ConMed, Johnson & Johnson, and Med Tech outside the submitted work. P.V.M. reports personal fees from ConMed. The other authors (I.A.F., R.E.P., S.T.N.M., D.A., Y.D.C.S., N.T.C., J.A.M.G., G.R.M.) declare that they have no known competing financial interests or personal relationships that could have appeared to influence the work reported in this article.
